# Multidrug-Resistant Tuberculosis, People’s Republic of China, 2007–2009

**DOI:** 10.3201/eid1710.110546

**Published:** 2011-10

**Authors:** Guang Xue He, Hai Ying Wang, Martien W. Borgdorff, Dick van Soolingen, Marieke J. van der Werf, Zhi Min Liu, Xue Zheng Li, Hui Guo, Yan Lin Zhao, Jay K. Varma, Christopher P. Tostado, Susan van den Hof

**Affiliations:** Author affiliations: Chinese Center for Disease Control and Prevention, Beijing, People’s Republic of China (G.X. He, H. Guo, Y.L. Zhao);; University of Amsterdam, Amsterdam, the Netherlands (G.X. He, M.W. Borgdorff, M.J. van der Werf, S. van den Hof);; Shandong Provincial Tuberculosis Control Center, Jinan, People’s Republic of China (H.Y. Wang, Z.M. Liu, X.Z. Li);; National Institute for Public Health and the Environment, Bilthoven, the Netherlands (D. van Soolingen);; KNCV Tuberculosis Foundation, The Hague, the Netherlands (M.J. van der Werf, S. van den Hof);; Centers for Disease Control and Prevention, Atlanta, Georgia, USA (J.K. Varma);; Tsinghua University, Beijing (C.P. Tostado)

**Keywords:** Tuberculosis and other mycobacteria, TB, Mycobacterium tuberculosis, M. tuberculosis, drug-resistant tuberculosis, multidrug-resistant tuberculosis, bacteria, MDR tuberculosis, MDR TB, genotype, genetic association studies, genotype-phenotype associations, cluster, clustering, antimicrobial resistance, People’s Republic of China, research

## Abstract

We conducted a case–control study to investigate risk factors for multidrug-resistant tuberculosis (MDR TB) in the People’s Republic of China. Genotyping analysis was used to estimate the percentage of cases from recent transmission among 100 MDR TB case-patients hospitalized during April 2007–July 2009. Molecular subtyping of isolates showed that 41% of MDR TB strains clustered. Beijing genotype was found in 94% of the MDR TB isolates and 79% of the pan-susceptible isolates. In multivariate analysis, MDR TB was independently associated with Beijing genotype, retreatment for TB, symptoms lasting >3 months before first evaluation at the hospital, lack of health insurance, and being a farmer (vs. being a student). MDR TB was associated with Beijing genotype and lower socioeconomic status. A large percentage of MDR TB cases seemed to result from recent transmission. Early detection, effective treatment, and infection control measures for MDR TB are needed to reduce transmission.

## Abstract 2

Early detection, effective treatment, and infection control measures are needed to reduce transmission.

Multidrug-resistant tuberculosis (MDR TB), defined as resistance to at least isoniazid and rifampin, has emerged as a global public health problem (*1*). The People’s Republic of China has the second greatest number of MDR TB cases in the world (*2*). According to the National Anti-Tuberculosis Drug Resistance Survey in 2007, an estimated 120,000 new MDR TB cases emerge annually in China, accounting for ≈24% of MDR TB worldwide (*3*). Although MDR TB represents only 8% of incident TB cases in China, controlling MDR TB is challenging because it is difficult to diagnose and treat (*4*). Thus, MDR TB is increasingly becoming a serious threat to TB control (*3*,*5*), and the recognition of extensively drug-resistant TB has furthered highlighted this threat (*6*,*7*).

The first pilot sites for the programmatic management of drug-resistant TB in China were established in October 2006. By the end of July 2010, similar management programs covered 41 prefectures/cities in 12 provinces in which ≈1,000 patients with MDR TB were treated with standardized treatment regimens recommended by the World Health Organization (*8*,*9*).

*Mycobacterium tuberculosis* acquires resistance to antimicrobial drugs through the selection of bacteria with mutations in resistance genes (*10*). Particular resistance genotypes, such as isoniazid-resistant strains from which the *katG* gene has been deleted, have been associated with decreased growth and persistence of *M. tuberculosis* in mice and guinea pigs (*11*). A recent molecular study suggests that drug-resistant strains of *M. tuberculosis* may be as transmissible as pan-sensitive strains (*12*). However, some isoniazid-resistant strains, such as those with a mutation at aa 315 of the *katG* gene, were as transmissible as drug-susceptible strains; these resistant, but equivalently transmissible, strains are typically associated with outbreaks (*13*–*15*).

In the past decade, many studies have evaluated the role of the Beijing genotype of *M. tuberculosis* in the worldwide TB epidemic (*16*,*17*). Beijing genotype strains are emerging in Southeast Asia, former Soviet republics, the Baltic states, and South Africa and are associated with multidrug resistance (*17*–*22*). In Europe, during 2003–2006, about half of MDR TB and extensively drug-resistant TB cases were caused by recent transmission, and 85% of those cases were caused by Beijing strains; during the same period, only 6%–7% of drug-susceptible TB cases in Europe were caused by Beijing strains (*21*,*22*). As the name suggests, Beijing genotype strains are particularly prevalent in China. In a survey of 10 provinces in China, the average percentage of Beijing genotype strains was 73%, but the percentage varied substantially by region, with the highest (93%) in the Beijing region (*23*).

Genotyping studies help elucidate transmission of TB by specific strains (*17*–*20*). Since 1993, IS*6110* restriction fragment-length polymorphism typing has been considered the standard for studying the molecular epidemiology of TB (*24*). Although restriction fragment-length polymorphism typing has brought significant new insights into TB transmission, the method is technically demanding and time-consuming (*23*). Therefore, a new standard typing method using mycobacterial interspersed repetitive unit–variable-number of tandem repeats (MIRU-VNTR) in the genome was recently proposed for studying clustering and transmission (*25*). The analysis of regions of difference (RDs) in the genome of *M. tuberculosis* complex can be used to study the phylogeny of these bacteria; this approach can also be used as an alternative to the more complicated spoligotyping method for Beijing genotype strain identification (*26*,*27*).

We used the RD105 deletion detection method to identify Beijing genotype strains. We also used 24-locus VNTR typing to investigate MDR TB transmission in patients admitted to the largest TB hospital in Shandong Province during April 2007–July 2009. Our goal was to characterize the genotypes of different MDR TB strains and identify specific risk factors associated with MDR TB and MDR TB strain clustering. A study in TB patients in the same hospital during 2004–2007 showed a prevalence of MDR TB of 10.8% (*28*). Although the national guideline of the TB control program requests directly observed treatment, in which TB patients take all doses under supervision, another study in rural Shandong showed that most TB patients do not receive directly observed treatment (*29*), which poses a risk for drug resistance. In Shandong Province, the programmatic management of drug-resistant TB has been introduced only in 1 prefecture, starting in October 2008.

## Methods

### Sampling Method

We conducted a case–control study at Shandong Provincial Tuberculosis Hospital (SPTH). We studied all 100 MDR TB patients and 97 patients infected with pan-sensitive TB strains who were admitted during April 2007–July 2009. The patients with pan-sensitive TB were randomly selected from an electronic database with information on 974 hospitalized patients. According to the national reporting system, ≈10% of all TB cases are reported by specialized TB hospitals.

### Data Collection

We obtained the following information from medical records for all study patients: sex, age, occupation, history of close contact with a TB patient (defined as a household member or colleague with TB), health insurance, TB treatment history, symptom duration before first evaluation at SPTH, presence of cavities on chest radiographs, current alcohol use, current smoking, sputum smear test results, and HIV status. During the study period, all enrolled patients were admitted to the hospital 1 time only, and the average admission time was ≈1 month.

### Drug Susceptibility Testing

Sputum samples from all hospitalized TB patients in SPTH were collected before the start of TB therapy and then cultured on Lowenstein-Jensen culture medium. *M. tuberculosis* complex was identified by culturing on Lowenstein-Jensen medium containing *p*-nitrobenzoic acid, where growth indicates that the bacilli are not part of the *M. tuberculosis* complex. Isolates from all patients with *M. tuberculosis*–positive culture results were subjected to drug susceptibility testing. The first-line drugs isoniazid, rifampin, streptomycin, and ethambutol were tested for drug susceptibility on the basis of the previously described proportion method (*30*). External quality assurance on drug susceptibility testing by proficiency testing is conducted regularly by the national reference laboratory of China and the supranational reference laboratory of the Public Health Laboratory, Hong Kong Special Administrative Region, China. In addition, results of drug susceptibility testing of study samples were rechecked by the national reference laboratory.

### DNA Extraction and Identification of Beijing Genotype TB Strain

DNA was extracted according to standard methods (*31*). The Beijing genotype TB strain was identified by using deletion-targeted multiplex PCR to detect the RD105 deletion (*26*,*27*). Primers were synthesized according to a study by Brosch et al. (*26*). The upstream primer used was 5′-GGAGTCGTTGAGGGTGTTCAGCTCAGCTCAGTC-3′; the 2 different downstream primers used were 5′-CGCCAAGGCCGCATAGTCACGGTCG-3′ and 5′-GGTTGCCCACTGGTCGATATGGTGGACTT-3′. An amplified sequence length of 761 bp corresponded to a Beijing genotype strain, and a sequence length of 1,466 bp corresponded to a non-Beijing genotype.

The reaction system comprised 1 µL each of upstream and downstream primer (0.4 μmol/L), 5 μL of 2× Taq PCR MasterMix (Tiangen Biotech [Beijing] Co., Ltd., Beijing, China), and 2 ng of DNA template with sufficient double-distilled water added to bring the final volume to 10 μL. Thirty-five cycles were applied in the PCR in the following manner. Each cycle consisted of denaturation at 95°C for 5 min, denaturation at 94°C for 30 s, annealing at 65°C for 30 s, followed by 72°C for 30 s, and then extension at 72°C for 7 min, after which products were stored at 4°C.

Amplification products were analyzed by using 1% agarose gel, stained by 1 μg/mL ethidium bromide in 1× TrIS-borate-EDTA electrophoresis buffer. The standard strain H37Rv was used as a control, and we used a 100-bp DNA ladder (Takara Biotechnology [Dalian] Co., Ltd., Dalian, China) for distinguishing molecular sizes.

### Genotyping

For genotyping, we used the 24-loci MIRU-VNTR method, as recommended by Supply et al. (*25*). Primers were designed as described (*25*) and synthesized by Shanghai Sangon Biological Engineering Technology and Service Co., Ltd. (Shanghai, China). Each PCR was performed with 2 ng of template DNA in a 20-μL final volume composed of 10 μL 2× PCR Master Mix (Tiangen Biotech [Beijing] Co., Ltd), 1 μL of each primer (final concentration 0.4 μmol/L), and 8 μL of distilled water.

Thermal cycling consisted of the first denaturation step at 95°C for 5 min, followed by 40 cycles of denaturation at 94°C for 50 s, annealing at 65°C for 50 s, and extension at 72°C for 1 min, with a final extension at 72°C for 5 min. PCR products were stored at 4°C before further analysis.

PCR products were analyzed by electrophoresis on 1% agarose gels after staining with ethidium bromide (1 μg/mL). The sizes of the products were determined by using a gel imager with a 100-bp DNA ladder (Takara) as a reference. The number of repeats at each locus was calculated in relation to the size of the locus. H37Rv was used as reference.

### Statistical Analyses

**Figure 1:**
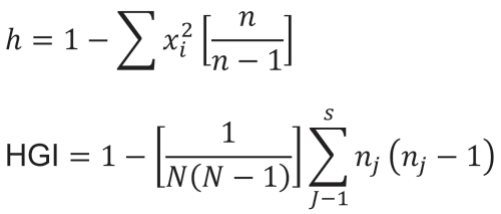
The allelic diversity of each VNTR loci (*h*) and the discriminating power according to the Hunter-Gaston index (HGI) were calculated by using this algorithm (*32*). MIRU-VNTR, mycobacterial interspersed repetitive unit–variable number of tandem repeats; *x_i_*, the frequency of allele *i*; *n,* total no. of strains in the scheme; *N,* total number of strains in the typing scheme; *s*, total no. of different MIRU-VNTR patterns, *n_j_*, no. of strains belonging to the *j^th^* type.

BioNumerics version 5.0 (Applied Maths, Inc., Sint-Martens-Latern, Belgium) was used to perform clustering and phylogenetic tree analyses. The allelic diversity of each VNTR loci (*h*) and the discriminating power according to the Hunter-Gaston index were calculated by using the algorithm shown in Figure 1.

Logistic regression analysis was done to compare MDR TB case-patients with pan-sensitive TB case-patients and to identify factors associated with clustering among MDR TB case-patients. We included the following variables in bivariate analysis: sex, age, occupation, history of close contact with a TB patient, health insurance, TB treatment history, symptom duration before first evaluation in SPTH, presence of cavity on chest radiograph, current alcohol use, current smoking, diagnostic sputum smear test results, HIV status, and Beijing genotype. Variables with a p value <0.2 were included in multivariable analyses. The final model was established by backward selection based on the fit of the model as tested with the likelihood ratio χ^2^ test (p = 0.05). All statistical analyses were performed by using SPSS version 17.0 (SPSS, Inc., Chicago, IL, USA).

### Ethical Issues

This research project was approved by the Chinese Ethical Committee for TB Operational Research, Chinese Center for Disease Control and Prevention, and SPTH. Patients were asked for written informed consent before drug susceptibility testing and genotyping were performed. Patients and TB hospitals were informed of test results, and the treatment regimen for each patient was adjusted according to the detected resistance profiles.

## Results

Among the 1,530 *M. tuberculosis* isolates from all hospitalized patients during the study period, 556 (36.3%) were resistant to >1 first-line drugs (isoniazid, rifampin, streptomycin, or ethambutol), of which 313 (20.5%) were resistant to 1 drug, 143 (9.3%) were resistant to >1 drug but were not MDR (poly-drug resistant), and 100 (6.5%) were MDR. A total of 33 of the 100 MDR TB patients and 47 of the 97 patients with pan-sensitive TB were from Jinan city, the capital of Shandong Province; other patients were from other parts of Shandong Province. The 97 selected controls were comparable to all patients with pan-sensitive TB with regard to potential confounding variables, such as age (38 vs. 37 years, p = 0.79), sex (69.1% vs. 70.0% male, p = 0.83), and retreatment (7.2% vs. 7.6%, p = 0.89).

### Genotyping Characteristics

The RD105 deletion test identified 94 of the 100 MDR TB strains and 77 (79%) of the 97 pan-sensitive strains as Beijing genotype strains (χ^2^ = 9.19, p = 0.002). Among 24 loci, MIRU-VNTR, QUB11b, Mtub21, and Mtub4 showed high allelic diversity (*h*>0.60), and MIRU26, QUB26, MIRU31, MIRU10, Mtub39, QUB4156, MIRU39, and ETR-A showed middle allelic diversity (*h*>0.30) (Table 1).

**Table 1:** Allelic diversity and number of repeats among 197 TB isolates, Shandong Province, People’s Republic of China, April 2007–July 2009*

Locus†	Allelic diversity	Allele no.	No. repeats, range
QUB11b	0.703	6	1–7
Mtub21	0.642	7	1–7
Mtub4	0.621	5	1–5
MIRU26	0.595	8	3–11
QUB26	0.551	9	2–10
MIRU31	0.494	7	2–9
MIRU10	0.340	4	1–4
Mtub39	0.335	7	1–7
QUB4156	0.331	5	1–5
MIRU39	0.326	3	2–4
ETR-A	0.303	7	1–7
Mtub30	0.295	4	2–5
MIRU40	0.263	6	1–6
MIRU4	0.237	6	0–7
MIRU16	0.175	4	1–4
MIRU27	0.156	3	1–3
ETR-B	0.146	3	1–3
MIRU20	0.146	3	1–3
MIRU23	0.093	4	2–6
Mtub34	0.073	2	2–3
Mtub29	0.055	4	2–5
ETR-C	0.055	3	2–4
MIRU2	0.000	1	1
MIRU24	0.000	1	1

*Allelic diversity and number of repeats was determined by using a 24-locus mycobacterial interspersed repetitive unit (MIRU)–variable-number of tandem repeats. TB, tuberculosis. †Loci are listed within each isolate group in descending order of allelic diversity.

**Figure 2:**
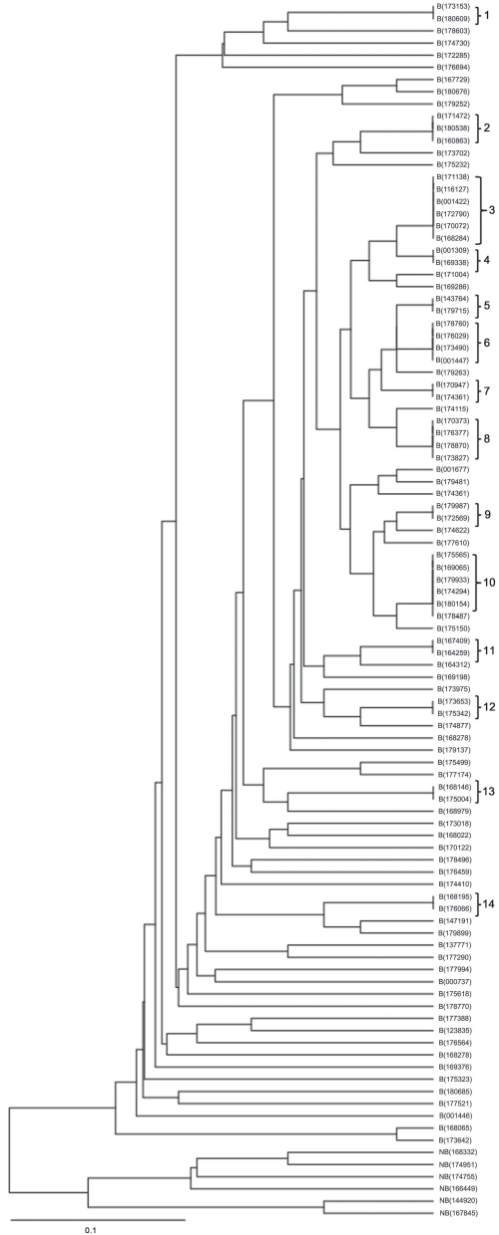
Dendrogram of 100 multidrug-resistant tuberculosis cases analyzed by using the RD105 deletion test and the MIRU-VNTR method. RD, region of difference; MIRU-VNTR, mycobacterial interspersed repetitive unit–variable number of tandem repeats; B, Beijing family; NB, no Beijing family. Scale bar indicates nucleotide substitutions per site.

We identified 73 different MIRU-VNTR genotype profiles among the 100 MDR TB strains, comprising 59 unique strains and 41 strains in 14 clusters; thus, 41% of the MDR TB cases clustered. The number of isolates in DNA fingerprint clusters ranged from 2 to 6. The discriminating power according to the Hunter-Gaston index method was 0.989. Dendrograms of 100 MDR TB cases, which were analyzed by using the RD105 deletion test and the MIRU-VNTR method, are shown in Figure 2. No clustering was detected among non–Beijing genotype cases.

### Determinants for Clustering among MDR TB Cases

In multivariate analysis comparing the 41 clustered MDR TB cases with the 59 nonclustered cases, we found that clustering was more likely to occur in isolates from patients who were <45 years of age (32 [50%] of 64) (adjusted odds ratio [OR] 3.5, 95% confidence interval [CI] 1.4–9.0), never previously treated (23 [51%] of 45; adjusted OR 2.6, 95% CI 1.1–6.2), and infected with Beijing genotype strains (41 of 100). Because the non–Beijing genotype strains did not include MDR TB strains, we did not calculate an OR.

### Determinants for MDR TB

Sixty-one percent of MDR TB case-patients were male; 49% were <35 years of age, and 55% were receiving retreatment for TB (Table 2). In bivariate analysis, MDR TB was not significantly associated with current alcohol use, current smoking, diagnostic sputum smear test results, or HIV status (tested patients all had negative results). Occupation, health insurance, TB treatment history, symptom duration before first evaluation in SPTH, cavity on chest radiographs, and genotype strain were significantly associated with MDR TB (p<0.05) (Table 2). Several of these factors remained significant in multivariate analysis, including previous TB treatment (adjusted OR 12.0, 95% CI 4.5–31.8), history of TB symptoms for >3 months before first visit to SPTH (adjusted OR 3.0, 95% CI 1.0–9.2), and lack of health insurance (adjusted OR 2.4, 95% CI 1.1–5.1). MDR TB occurred less frequently in students (adjusted OR 0.2, 95% CI 0.1–0.7) than farmers. MDR TB cases were also more likely to be caused by a Beijing genotype TB strain (adjusted OR 4.3, 95% CI 1.4–13.9).

**Table 2:** Risk factors for MDR TB, Shandong Province, People’s Republic of China, April 2007–July 2009*

Patient data	No. (%) patients with MDR TB, n = 100	No. (%) patients with pan-sensitive TB, n = 97	Crude OR (95% CI)	Adjusted OR† (95% CI)
Sex				
M	61 (61.0)	67 (69.1)	1	
F	39 (39.0)	30 (30.9)	0.7 (0.4–1.3)	
Age group, y				
15–34	49 (49.0)	56 (57.7)	1	
35–54	38 (38.0)	21 (21.6)	2.1 (1.1–4.0)	
>55	13 (13.0)	20 (20.6)	0.7 (0.3–1.6)	
Occupation				
Farmer	39 (39.0)	29 (29.9)	1	1
Blue-collar worker	16 (16.0)	12 (12.4)	1.0 (0.4–2.4)	0.8 (0.2–2.4)
White-collar worker	37 (37.0)	39 (40.2)	0.7 (0.4–1.4)	0.6 (0.3–1.4)
Student	8 (8.0)	17 (17.5)	0.4 (0.1–0.9)	0.2 (0.1–0.7)
Close contact with TB patient				
No	90 (90.0)	90 (92.8)	1	
Yes	10 (10.0)	7 (7.2)	0.7 (0.3–1.9)	
Health insurance				
Yes	33 (33.0)	48 (49.5)	1	1
No	67 (67.0)	49 (50.5)	2.0 (1.1–3.5)	2.4 (1.1–5.1)
History of TB treatment				
New case	45 (45.0)	90 (92.8)	1	1
Retreatment case	55 (55.0)	7 (7.2)	15.7 (6.6–37.3)	12.0 (4.5–31.8)
Symptom duration before first evaluation in Shandong Provincial TB Hospital, mo				
<1	14 (14.0)	31 (32.0)	1	1
1–2	16 (16.0)	30 (30.9)	1.2 (0.5–2.8)	1.3 (0.5–3.8)
3–5	14 (14.0)	17 (17.5)	1.8 (0.7–4.7)	3.0 (1.0–9.2)
>6	56 (56.0)	19 (19.6)	6.5 (2.9–14.8)	3.3 (1.2–9.1)
Cavity visible on radiograph				
No	55 (55.0)	67 (69.1)	1	
Yes	45 (45.0)	30 (30.9)	1.8 (1.0–3.3)	
Beijing genotype				
No	6 (6.0)	20 (20.6)	1	1
Yes	94 (94.0)	77 (79.4)	4.1 (1.6–10.6)	4.3 (1.4–13.9)

*MDR TB, multidrug-resistant tuberculosis; OR, odds ratio; CI, confidence interval. †Adjusted for all other factors included in the multivariate model.

## Discussion

In this study in Shandong Province, the fact that 45% of new TB cases are MDR TB and the high percentage of clustering among isolates (51%) indicate substantial MDR TB transmission. The percentage of case clustering among persons previously treated for MDR TB was also substantial (33%). Thus, a large percentage of MDR TB cases in Shandong Province most likely were caused by recent transmission. Nosocomial transmission is not likely to explain the high percentage of clustering because sputum samples were obtained at hospital admission. MDR TB was more likely to be diagnosed in population groups of lower socioeconomic status, such as farmers, persons with low education level, and those without health insurance. These findings suggest that the prevalence of MDR TB among TB case-patients may be poverty related; therefore, these lower socioeconomic groups should be the highest priority for MDR TB prevention efforts.

Our molecular analysis found a high discriminative power for using 24-locus MIRU-VNTR genotyping. We found that 41% of MDR TB isolates shared identical VNTR profiles, and clusters of up to 6 cases in size were observed. In general, MDR TB has a low conversion rate and lengthy negative conversion period, given its high capacity for transmission (*9*). This phenomenon indicates that some MDR TB strains might give rise to widespread transmission of MDR TB.

Our genotyping analysis showed that strain clustering was more common among young patients, in new cases, and in TB caused by Beijing genotype strains. The finding regarding younger patients was in agreement with a previous study, suggesting that TB among young patients more frequently is clustered (*33*).

Most MDR TB cases occurred among patients who were previously treated, were less educated, lacked health insurance, or had a medical history of TB for >3 months before entering SPTH. Patients who have previously received treatment are at risk for MDR TB because of inadequate treatment of drug-sensitive TB or because of relapse after a previous episode of unrecognized MDR TB that was incorrectly treated as drug sensitive. Although reinfection also should always be considered (*34*), our findings reaffirm the need to prevent MDR TB through effective anti-TB drug regimens that are based on drug susceptibility profiles and are administered under direct supervision for the correct duration. Efforts are needed to strengthen basic TB diagnosis and treatment at the primary health care level, where such patients are likely to first contact the health system.

Beijing genotype strains are more common in Asia, former Soviet republics, and South Africa, but they are spreading worldwide (*35*,*36*). Several studies have shown that these strains are prevalent in China, especially northern China (*23*). A previous study found that non-Beijing strains represent 20.6% of strains in Shandong Province (*37*). In our sample from Shandong Province, Beijing genotype strains constituted 94% of MDR TB and 80% of pan-sensitive cases, suggesting that Beijing genotype is associated with MDR TB phenotype. Drobniewski et al. suggested that these Beijing strains have greater virulence and transmissibility and a tendency toward generation of MDR TB, although the mechanism remains unclear (*38*).

Our study has 2 main limitations. First, patients in this study were recruited only from 1 large TB hospital. The clustering proportion will depend on the completeness of the sampling, in which increasing sampling fractions will identify more clustering (*39*,*40*). Thus, our results likely underestimate the percentage of clustering. Because no reliable estimates exist of the incidence and prevalence of MDR TB in Shandong Province, we do not know the percentage of MDR TB case-patients who were hospitalized in the study hospital and thus cannot estimate the extent to which we underestimated the clustering percentage. Studying a larger, geographically defined population will help us better understand the transmission dynamics of MDR TB and further identify populations or settings that should be made a priority for MDR TB prevention. Second, we did not investigate epidemiologic links between patients, such as home, job site, community, or congregate settings.

MDR TB presents one of the major challenges in TB control in China (*3*,*30*). Our findings of a relatively high prevalence of MDR TB among new TB cases and a high percentage of clustering among MDR TB cases show that MDR TB transmission is a large problem in Shandong Province, China, highlighting the risk for further occurrence of drug-resistant TB. The general programmatic management of MDR TB as well as specific measures are urgently needed to control transmission of MDR TB in China.

Our study highlights the challenges of controlling MDR TB in China. There is a trend toward lower risk for MDR TB in higher socioeconomic groups. The recent transmission of MDR TB may be contributing to a large percentage of cases. Without early diagnosis and effective treatment, these new cases will continue to generate further infections in the community. China must urgently scale up prevention of MDR TB through better infection control, enhancement of directly-observed treatments, universal use of appropriate diagnosis and treatment for MDR TB. Similarly, Beijing strains continue to predominate and to be associated with MDR TB, meaning that more dedicated research is needed to understand why these strains have survived and thrived in China, as well as other countries.
